# Lifelong Learners’ Contributions to the Campus Community

**DOI:** 10.1093/geroni/igaa057.1817

**Published:** 2020-12-16

**Authors:** Barbara White

**Affiliations:** California State University, Long Beach, Huntington Beach, California, United States

## Abstract

The 2,000 member Osher Lifelong Learning Institute (OLLI) at California State University, Long Beach offers non-credit classes to adults 50 and older. We have an ongoing strategic goal (2014, 2019) to “increase our University and student involvement.” Integration into the campus community includes members volunteering as participants in survey and participatory faculty and student research related to biopsychosocial aspects of aging, faculty/student data collection in selected OLLI classes, vetting students to teach OLLI classes, acting as resources for professors/students in product development related to aging, guest lecturing in University courses, and providing internships for students at OLLI. Collaborations have led to multiple faculty/student publications and presentations. We also endow an annual award for graduate students to support their research/projects related to aging. In return, we request and provide students the opportunity to present their results to our members. Outreach strategies will be discussed.

